# Risk Assessment on Benzene Exposure among Gasoline Station Workers

**DOI:** 10.3390/ijerph16142545

**Published:** 2019-07-16

**Authors:** Sunisa Chaiklieng, Pornnapa Suggaravetsiri, Herman Autrup

**Affiliations:** 1 Department of Environmental Health, Occupational Health and Safety, Faculty of Public Health, Khon Kaen University, Khon Kaen 40002, Thailand; 2 Department of Epidemiology and Biostatistics, Faculty of Public Health, Khon Kaen University, Khon Kaen 40002, Thailand; 3 Institute of Public Health, University of Aarhus, 8000 Aarhus, Denmark

**Keywords:** benzene, cashier, cancer risk, fueling, human health risk

## Abstract

Benzene is a human carcinogen presented in gasoline (1% by volume). It is also found in vehicle exhaust. The aim of this study was to assess the health risk of inhalation exposure to benzene among gasoline station workers. The ambient benzene concentration was measured by personal sampling from 150 gasoline station workers (137 fueling workers and 13 cashiers). Additional data of working characteristics were collected by interviews and on-site observations. All workers were non-smokers and passive smoking was limited. Risk assessment of inhalation exposure was determined using the United State Environmental Protection Agency (USEPA), and showed a high risk of adverse health effect (Hazard Quotients (HQ) >1) in 51.33% of workers. The cancer risk was increased from 1.35 × 10^−8^ to 1.52 × 10^−4^, and 70.67% of the workers had a lifetime cancer risk (>Inhalation Unit Risk (IUR): 2.2 × 10^−6^). A significantly higher risk was found in fueling workers compared to cashiers, and in workers at gasoline stations in inner-city zones (suburban and urban), compared to rural zones. All risk estimations were based upon a single measurement in an eight hour working period, which was assumed to be the average shift length for all working days in a year (250 days). The increased health risk suggests that there should be health surveillance for workers in order to protect them from exposure to benzene. In addition to benzene, the volatile organic compounds (VOCs) present in gasoline may influence health outcomes.

## 1. Introduction

Benzene is a group 1 human carcinogen classified by International Agency for Research on Cancer (IARC) [1]. Entry into the human body can occur orally through eating, dermal contact, and respiration, chronic exposure can cause adverse health effects and affect the blood circular system (anemia, leukopenia, and thrombocytopenia) [2]. The Bureau of Epidemiology, Thailand [3] reported that, among the 78 cases of petroleum toxicity, benzene toxicity accounted for 12 cases. Respiration is the main route of benzene exposure from ambient air [4].

The National Institute Occupational Safety and Health (NIOSH) recommends an occupational exposure limit (OEL) of 0.1 ppm [5], which is the recommended exposure limit (REL) using an 8 hour time weighted average (TWA) that should not cause harm to the worker’s health. A previous study indicated that most of the benzene pollution is from traffic released in vehicle exhaust or where gasoline is stored, such as in refineries. A higher concentration of benzene than the OEL standard was previously found in the ambient air of refinery environments that exhibits a health risk for workers but depends on the exposure time period per day [6]. A study confirmed that the highest ambient concentration of benzene was found at gasoline stations compared to other sites in public areas e.g., schools, public transport stations [7]. Recent studies found benzene in the ambient air of public areas such as major roads with heavy traffic [8], urban areas [9], or school areas in urban environments [7], to be lower than 0.1 ppm. These concentrations could result in an increased health risk [8]. Worker’s health risk profiles from benzene exposure at gasoline stations in Thailand has not previously been studied.

The number of car registrations and the quantity of gasoline/petrol sold is increasing in Thailand each year. Khon Kaen, a provincial city in Northeast of Thailand, has the top sales record of gasoline [10]. Benzene was included as a one percent component of gasoline in Thailand [10]. Our previous study found that the ambient concentrations of benzene at gasoline stations were location specific, dependent on the quantity of gasoline sold and personal job profile, and were lower than 0.1 ppm [11]. A later study showed that inhalation of ambient benzene throughout a working period without proper protection could result in adverse effects related to benzene toxicity [12]. Our pilot study showed that even at low exposures to benzene, some gasoline station workers had an increased carcinogenic risk based on long term exposure [13]. A health risk analysis from benzene exposure has previously shown that people living in urban areas had a significantly higher cancer risk than those living in rural areas [7]. An accumulation of other volatile organic compounds (VOCs) could also affect the magnitude of human health risk [14]. Personal time spent active or daily exposure hours at a worksite was one of the factors considered in carcinogenic risk assessment from benzene exposure following the United State Environmental Protection Agency (USEPA)-Integrated Risk Information System (IRIS)’s guidance [4,13].

Our previous study considered the ambient benzene concentration at gasoline stations. However, personal benzene exposure from job functions and gasoline station location could not demonstrate the risk profile among workers with different job functions, at different station locations and with different lengths of time of exposure. This study aimed to assess the health risk following the USEPA-IRIS risk assessment protocol on inhalation exposure to benzene at different locations and among workers with different job functions at gasoline stations.

## 2. Materials and Methods

### 2.1. Sampling Site and Study Population

**Figure 1:**
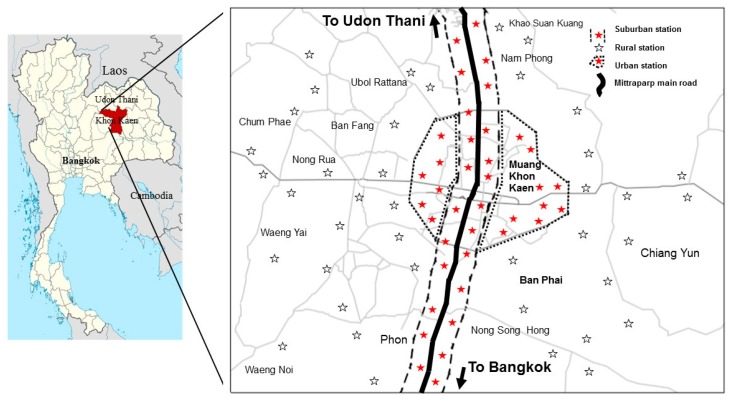
Map of Khon Kaen, the study site of the gasoline stations.

This study was conducted in the city of Khon Kaen, Thailand and included 98 gasoline stations. as shown in a map (Figure 1). The sample size was calculated by stratified random sampling [15]. Locations or areas of gasoline stations were categorized into three zones as ’urban’, ‘suburban’ and ‘rural’. The individual variances of each zone were based on concentrations from the study of Lekcharernkul et al. [16], the minimum requirement of sample size was 98. The gasoline stations in the urban zone were defined as those located in the Nai Muang subdistrict of the city of Khon Kaen and where the majority of the residents occupations were not in agriculture; the suburban gasoline stations were located around the Nai Muang subdistrict and near the main highway, Mittraparp road, which connects the provincial city of Khon Kaen to the capital city of Bangkok. The rural gasoline stations were those located outside the Nai Muang subdistrict and where the majority of the residents worked in agriculture. There were 150 gasoline workers included into the study, representative of the different stations, zones, and job functions (cashier or fueling) of workers.

Additional data of working characteristics were collected via subject interviews and observation. All workers were non-smokers and passive smoking was limited. All participants from the previous study had informed consent before entering into this study, which was approved by Khon Kaen University Ethics Committee in human research no. HE562237 and HE612102.

### 2.2. Air Sampling and Analysis for Benzene Concentration

Air benzene monitoring was done by personal sampling with an active sampler with a low flow rate control pump and using a coconut charcoal sorbent tube following the standard method of National Institute Occupational Safety and Health (NIOSH) number 1501 [17]. The sampling was carried out during the dry season (November–April) of Northeast Thailand. The single measurement by personal sampling was done in the 8 hour working period of each worker. Temperature ranged from 21.9 °C to 35.5 °C, humidity was 52% to 94.6%, and the wind velocity range was 0.63 to 5.75 km/hour. Benzene concentration was analyzed by gas chromatography with flame ionization detector (GC-FID) (Hewlett Packard 1996, Germany).

### 2.3. Risk Assessment

Benzene intake though inhalation was calculated following the United State Environmental Protection Agency’s (USEPA) [18] conditions for occupational exposure. Exposure duration (ED), and exposure frequency (EF) values were derived from interview data and the inhalation uptake of 50% of all intake (exposure concentration (EC)) [4]. 

Exposure was done by calculation of inhalation intake (EC) at concentration of inhaled air benzene as the following formula:

EC = exposure concentration or intake (µg/m^3^)

EC = (CA × ET × EF × ED)/AT

CA = benzene concentration (µg/m^3^)

ET = exposure time, hours/day = 8 hours/day or longer exposure time depending on individual data of workers

EF = exposure frequency (5 days/week × 50 weeks/year) = 250 days/year guided by the USEPA [19]

ED = exposure duration (25 years)

AT = averaging time = average time in hours per exposure period (25 years for general working period is equivalent to 219,150 hours and 70 years for lifetime cancer risk characterization (70 years × 365 days/year × 24 hours/day = 613,200 hours) guided by the USEPA [18]

Cancer risk characterization by comparison to Inhalation Unit Cancer Risk (IUR) was considered as the following;

Cancer risk = IUR × EC

Where IUR = 2.2 × 10^−6^ to 7.8 × 10^−6^ per 1 µg/m^3^ [4]

If the risk value > IUR or 2.2 × 10^−6^ to 7.8 × 10^−6^, that means an unacceptable risk concerning cancer [20]

If the risk value < 2.2 × 10^−6^, that means an acceptable risk of cancer

Non-cancer risk assessment is considered as the Hazard Quotients (HQ) calculation of non-cancer risk from chronic exposure to inhaled benzene was done by following the USEPA [18].

HQ (unitless) = EC/RfC

EC (µg/m3) = exposure concentration in air

Reference concentration (RfC) of benzene is 0.03 mg/m^3^ from the USEPA-IRIS [4].

If HQ > 1, that means the risk is unacceptable, if HQ < 1, that means the risk is acceptable/

### 2.4. Statistics Analysis

Data were analyzed by STATA version 10 software. Frequency of exposure and concentration level of benzene were described. The value of risk estimation was described from the perspective of the personal benzene concentration from each worker, the average concentration, and the 95th percentile of the concentration values. The inferential statistic was a student t-test for the difference of means of cancer risk (>IUR) and non-cancer risk (HQ > 1) between zones and job functions of workers. The statistical significance was set at *p*-value <0.05.

## 3. Results

### 3.1. Benzene Concentrations and Benzene Exposures

Of 150 gasoline station workers, there were 82 males (54.67%) and 68 female workers (45.33%) who participated in the study. The job function was classified into two groups; 137 fueling workers and 13 cashiers. For the location of the stations, 48 workers (32%) were from an urban zone, 60 workers (40%) from a sub-urban zone and 42 workers (28%) from a rural zone. The benzene concentration in inhaled air ranged from 0.03 to 65.71 parts per billion (ppb) as shown in Table 1.

The exposures from each measured value were estimated to be representative for all working days in a year (250 days). In general, shift length was 8 hours, however, the shifts were different between stations with a range of 8–17 hours/day. To estimate the inhalation intake at the 95th percentile of concentration (CA = 0.049 ppm or 159.08 µg/m^3^), the following variables were used: ET: 8 hours/day or more depending upon individual work characteristics (variable data); EF: 250 days/year; ED: 25 years; and AT, which was divided into two cases, cancer risk: 70 years × 365 days/year × 24 hours/day = 613,200 hours, and non-cancer risk: 25 years × 365 days/year × 24 hours/day = 219,000 hours. For example, using these variables and a benzene concentration of 49 ppb, the potential intake for cancer risk is 12.97 µg/m^3^ and 0.036 mg/m^3^ for non-cancer risk. The potential intakes were calculated based on personal inhaled benzene concentration (individual), the 50th percentile of concentration values, and the 95th percentile of concentration values. The daily work hour period of the individual was considered for the inhalation intake. The potential risk assessment for non-cancer risk from benzene exposure was also indicated as a condition of 50% inhalation uptake of benzene from inhaled air [4].

### 3.2. Lifetime Cancer Risk of Gasoline Station Workers Classified by Zone of Stations

The lifetime cancer risk across all zones showed a range of average cancer risk between 1.4 × 10^−5^ and 8.0 × 10^−5^ which caused an unacceptable lifetime cancer risk according to IUR (>2.2 × 10^−6^) [4,20]. The individual cancer risk estimation showed the worst cases of cancer risk (1.5 × 10^−4^) in the inner-city zone of Khon Kaen (urban and suburban areas). The unacceptable lifetime cancer risk affected 70.67% of gasoline workers (106 workers). The lifetime cancer risk of workers was significantly different among three zones (*p*-value = 0.007) as shown in Table 2. Based on inhalation uptake of benzene concentration of inhaled air at the 95th percentile, the lifetime cancer risk across all zones was from 2.1 × 10^−5^ to 1.5 × 10^−4^. Inhalation exposure to benzene concentration below OEL caused unacceptable risk of cancer among gasoline station workers.

### 3.3. Lifetime Cancer Risk of Gasoline Station Workers Classified by Job Functions

The lifetime cancer risk classified by job function showed the highest risk of cancer in fueling workers (1.5 × 10^−4^). All fueling workers and cashiers had potential cancer risk when considering the 95th percentile of benzene concentrations (0.049 ppb). The average of individual lifetime cancer risk of fueling workers from benzene exposure (>2.2 × 10^−6^) was 6.7 × 10^−5^, which was significantly higher than that of cashiers (1.1 × 10^−5^) at *p*-value < 0.001 (Table 3).

### 3.4. Non-Cancer Risk of Gasoline Station Workers Classified by Zone of Stations and Job Functions

The health risk estimation showed that based on chronic inhalation, benzene exceeded the safety reference value (RfC = 0.03 mg/m3) [4] in 51.33% of the workers. For individual risk estimation, the hazard quotient (HQ) range was from 5.7 × 10^−4^ to 1.82. The highest risk found in the suburban zone was significantly different from the other zones at *p*-value = 0.024 (Table 4). Furthermore, fueling workers had significantly higher risk than cashiers (Table 5). Considering 50% inhalation uptake of benzene from inhaled air, the average HQ across all zones indicated an acceptable risk for non-carcinogenic effects (HQ = 0.45–0.91).

## 4. Discussion

### 4.1. Lifetime Cancer Risk of Gasoline Station Workers

The benzene concentrations at gasoline stations in this study ranged from 0.03 to 65.71 parts per billion (ppb) which did not exceed the OEL (100 ppb) recommended by NIOSH [5]. However, fifty percent of workers exceeded 50% of the OEL and this may play an important role in the higher than acceptable health risk these workers face [12,13]. The concentration was lower than a study at gasoline stations in Bangkok (107.68 ppb) [21]. The explanation might be that the traffic volume in Khon Kaen province is low compared to Bangkok, in particular the number of motor bikes is very high in certain areas of Bangkok [22]. Furthermore, the emission may be diluted due to an open landscape. Based on an inhalation uptake, daily exposure to benzene at the 95th percentile concentration (0.049 ppb) affected the lifetime cancer risk of the Khon Kaen workers across all zones and functions which was consistent with the study in Bangkok, Thailand [21]. 

Our study showed that the benzene exposure potentially increased the cancer risk for 70.67% of workers, with a significant difference in the risk among the three zones. The highest cancer risk of about one in ten thousand workers (1.5 × 10^−4^) was found among gasoline workers of the inner city (urban and suburban zones). A similar study in a bus depot in central Johannesburg, South Africa also showed that the work location was associated with an increased cancer risk for fuel pump attendants [23]. Another study in Turkey confirmed that workers in the dispensing area of gasoline stations had a cancer risk from long-term exposure to benzene of one in 10,000 people (1.3 × 10^−4^) [7]. This is consistent with our finding, that the highest cancer risk of about one in 10,000 workers was also indicated in the suburban zone. That might be explained by the locations of petrol stations along the main highway, Mittraparp Road, which connects Bangkok and some Mekong sub-region countries and has heavy traffic in comparison to a rural area without a highway [11]. A contribution of benzene from car exhaust emission has previously confirmed that people living in urban areas are more likely to be affected by cancer risk than those in rural areas [8]. 

In a similar study, workers in oil refineries who had a higher cancer risk (3.4 × 10^−6^) than office workers (2 × 10^−6^) in the same company [24], similar to fueling workers who were at a higher risk than the cashiers. Higher exposures to benzene of petrol station workers were detected by using urinary t,t-muconic acid, a biomarker of benzene exposure, when compared to taxi drivers [25]. Moreover, workers operating fuel dispensers might be close to the source of benzene vapor, increasing benzene exposure as previously reported among maintenance workers of petrochemical plants during maintenance operations [26].

### 4.2. Non-Cancer Risk of Gasoline Station Workers

There is no clear evidence for adverse health effects from low benzene exposure and an acceptable risk (HQ < 1) was already reported, for example in urban areas in China [9] and in Bangkok traffic areas [21]. However, these studies are in contrast to the study where workers at fuel stations reported higher than acceptable health risks from benzene exposure [12]. This is similar to our study which has shown that the health risk of chronic effects was indicated in 51.33% of gasoline station workers. Moreover, a significant difference was demonstrated among the three zones. This is consistent with previous studies where the health risk of benzene exposure was higher in industrial areas compared to residential areas [26,27]. Regarding 50% inhalation uptake of benzene exposure, the non-carcinogenic health risk from benzene exposure was not significantly shown among workers in Khon Kaen gasoline stations. Our study limitation is that only airborne benzene concentration was measured. Further investigations should also focus on biomarker monitoring for early detection of benzene exposure [28].

The significantly higher risk for adverse health effects of fueling workers compared to the cashiers supports the previous report that fueling was associated with the highest risk of benzene exposure out of all job functions for gasoline station workers [13]. The study of oil refineries showed that oil refiners had a higher risk than office workers [21]. In addition, a study showed that security guardsmen, motorcycle drivers, and street vendors all had lower health risks than refueling workers [29]. Moreover, the previous study showed that professional car drivers were more likely to be at risk from exposure to benzene than passengers due to much longer hours of exposure to the same concentration [30]. This supported the results of our study that the job function also played a role in the effect of benzene exposure per hour worked on health risk assessment. 

## 5. Conclusions

Based on an estimation of human health risk using the inhalation scenario model based upon the USEPA-IRIS, the results showed that chronic exposure to benzene concentrations below the NIOSH-OEL could also exhibit a cancer risk of >2.2 × 10^−6^ in 70.67% of gasoline workers. Fueling workers had a significantly higher lifetime cancer risk than cashiers. Workers in gasoline stations in the suburban zone showed a significantly higher risk than other zones. For non-carcinogenic effects, the HQ was exceeded in 51% of workers who were fueling, they had a higher than acceptable risk of adverse health effects from low benzene exposure. With an inhalation uptake of benzene in inhaled air of 0.049 ppm, the increased risk of cancer was exhibited across all zones and functions of gasoline station workers in this study. Therefore, an occupational health regulation and surveillance program is recommended for gasoline station workers. Moreover, the optimal the 8 hours per day at the worksite and good practice of occupational safety and health protection to reduce benzene exposure should be observed while dispensing fuel. Further studies on the risk assessment of VOCs and carcinogen exposure in the hazardous zones at gasoline stations are of interest.

## Figures and Tables

**Table 1:** Concentration of benzene classified by zones and functions of gasoline station workers (ppm).

Zone/Function	Mean (SD)	Median	95th Percentile	95% CI
**Zone**				
- Urban (48)	0.024 (0.023)	0.012	0.049	0.017–0.030
- Suburban (60)	0.035 (0.021)	0.049	0.049	0.030–0.041
- Rural (42)	0.023 (0.024)	0.013	0.049	0.016–0.030
**Job function**				
- Fueling (137)	0.031 (0.022)	0.050	0.049	0.027–0.035
- Cashier (13)	<0.001 (0.002)	<0.001	0.006	−0.0005–0.001

Limit of detection = 0.00003 ppm or 0.03 ppb.

**Table 2:** The average lifetime cancer risk classified by zone.

Concentration Value Used	Cancer Risk of Workers in Different Zones		
	Urban (n = 48)	Suburban (n = 60)	Rural (n = 42)
Individual ^1^	1.8 × 10^−5^–5.1 × 10^−5^	1.9 × 10^−5^–8.0 × 10^−5^	1.4 × 10^−5^–4.7 × 10^−5^
(Min–Max)	1.8 × 10^−8^–1.5 × 10^−4^	1.4 × 10^−8^–1.5 × 10^−4^	1.8 × 10^−8^–1.3 × 10^−4^
50th Percentile	1.4 × 10^−5^–4.9 × 10^−5^	2.2 × 10^−5^–7.9 × 10^−5^	1.3 × 10^−5^–4.7 × 10^−5^
(Min–Max)	1.0× 10^−5^–7.2 × 10^−5^	1.5 × 10^−5^–1.1 × 10^−4^	1.3 × 10^−5^–5.8 × 10^−5^
95th Percentile	2.9 × 10^−5^–1.0 × 10^−4^	3.1 × 10^−5^–1.1 × 10^−4^	2.9 × 10^−5^–1.0 × 10^−4^
(Min–Max)	2.1 × 10^−5^–1.5 × 10^−4^	2.1 × 10^−5^–1.5 × 10^−4^	2.9 × 10^−5^–1.3 × 10^−4^

^1^ Significant difference of individual cancer risk among three zones at *p*-value = 0.007. The individual, 50th percentile, and 95th percentile means used personal benzene concentration, average concentration, and 95th percentile of concentrations, respectively for cancer risk estimation. Inhalation Unit Risk (IUR) of cancer risk is >2.2 × 10^−6^–7.8 × 10^−6^.

**Table 3:** The average lifetime cancer risk classified by job functions.

Cancer Risk	* Cancer Risk Among Job Functions	
	Fueling (n = 137)	Cashier (n = 13)
Individual ^1^	1.9 × 10^−5^–6.7 × 10^−5^	3.3 × 10^−6^–1.1 × 10^−5^
(Min–Max)	1.4 × 10^−8^–1.5 × 10^−4^	1.8 × 10^−8^–1.0 × 10^−4^
50th Percentile	1.7 × 10^−5^–6.0 × 10^−5^	2.9 × 10^−7^–1.0 × 10^−6^
(Min–Max)	1.2 × 10^−5^–8.6 × 10^−5^	2.2 × 10^−7^–1.2 × 10^−6^
95th Percentile	3.0 × 10^−5^–1.1 × 10^−4^	3.5 × 10^−6^–1.2 × 10^−5^
(Min–Max)	2.1 × 10^−5^–1.5 × 10^−4^	2.6 × 10^−6^–1.4 × 10^−5^

^1^ Significant difference of individual cancer risk between fueling and cashier at *p*-value < 0.001. The individual, 50th percentile, and 95th percentile means used personal benzene concentration, average concentration, and 95th percentile of concentration of benzene, respectively for cancer risk estimation. * IUR of cancer risk is >2.2 × 10^−6^–7.8 × 10^−6^.

**Table 4:** Non-cancer risk presented by hazard quotient (HQ) classified by zone (n = 150).

Zone	Hazard Quotient					
	Individual HQ ^1^		HQ 50th Percentile ^2^		HQ 95th Percentile	
	Range	Mean	Range	Mean	Range	Mean
Urban (n = 48)	5.71 × 10^−4^ 1.82	0.61	0.43–0.86	0.59	0.91–1.82	1.24
Suburban (n = 60)	5.71 × 10^−4^ 1.82	0.96	0.65–1.29	0.94	0.91–1.82	1.32
Rural (n = 42)	7.61 × 10^−4^ 1.51	0.56	0.56–0.70	0.57	1.21–1.51	1.23

^1^ Significant difference of individual HQ among three zones at *p*-value < 0.05; ^2^ Significant difference of HQ 50th percentile among three zones at *p*-value < 0.05; The individual, 50th percentile, and 95th percentile means used personal benzene concentration, average concentration, and 95th percentile of concentrations, respectively for non-cancer risk estimation.

**Table 5:** Non-cancer risk presented by hazard quotient (HQ) classified by job function (n = 150).

Job Function	Hazard Quotient					
	Individual HQ ^1^		HQ 50th Percentile		HQ 95th Percentile	
	Range	Mean	Range	Mean	Range	Mean
Fueling (n = 137)	5.71 × 10^−4^–1.82	0.80	0.51–1.03	0.72	0.91–1.82	1.28
Cashier (n = 13)	5.71 × 10^−4^–0.16	0.01	0.01–0.01	0.01	0.11–0.16	0.15

^1^ Significant difference of individual HQ between fueling and cashier at *p* < 0.001; The individual, 50th percentile, and 95th percentile means used personal benzene concentration, average concentration, and 95th percentile of concentrations, respectively for non-cancer risk estimation.
